# A comparison of phage susceptibility testing with two liquid high-throughput methods

**DOI:** 10.3389/fmicb.2024.1386245

**Published:** 2024-08-07

**Authors:** Krupa Parmar, Joseph R. Fackler, Zuriel Rivas, Jay Mandrekar, Kerryl E. Greenwood-Quaintance, Robin Patel

**Affiliations:** ^1^Division of Clinical Microbiology, Department of Laboratory Medicine and Pathology, Mayo Clinic, Rochester, MN, United States; ^2^Adaptive Phage Therapeutics, Inc. (APT), Gaithersburg, MD, United States; ^3^Division of Biomedical Statistics and Informatics, Department of Health Sciences Research, Mayo Clinic, Rochester, MN, United States; ^4^Division of Public Health, Infectious Diseases, and Occupational Medicine, Department of Medicine, Mayo Clinic, Rochester, MN, United States

**Keywords:** phage therapy, phage susceptibility testing, assay reproducibility, *Staphylococcus aureus*, *Pseudomonas aeruginosa*

## Abstract

Phage therapy is a promising antibacterial strategy, especially given that drug-resistant bacterial infections are escalating worldwide. Because phages are not active against all strains of a given species, phages being considered for therapeutic use would ideally be tested against bacterial isolates from individual patients prior to administration. Standardized, clinically validated phage susceptibility testing (PST) methods are needed for assessing *in vitro* phage activity. This study compared two high-throughput liquid-based PST assays. The first, using the Biolog Omnilog^™^, assessed changes in microbial respiration leading to color changes based on a tetrazolium dye. The second, Agilent BioTek Cytation 7, assessed changes in optical density. Both used 96-well microtiter plate formats. A total of 55 diverse phages with activity against *Escherichia coli*, *Staphylococcus aureus*, *Pseudomonas aeruginosa*, *Acinetobacter baumannii*, or *Enterococcus faecalis* were studied against their respective susceptible bacterial hosts and non-susceptible controls, with susceptibility defined based on plaque assay. PST was performed by both assays in replicates, with results compared in terms of hold times (time through which bacterial growth is inhibited by phage compared to controls). Coefficients of variance and interclass correlation coefficients were used to assess inter- and intra-assay reproducibility. Based on a ≤50% coefficient of variance cutpoint, 87% of Biolog and 84% of Agilent assays were considered valid for susceptible bacteria, with 100% considered valid for non-susceptible bacteria by both systems. Using a 8 h hold time cutpoint, 100% of the results matched between the two assays. The interclass correlation coefficient showed 26% excellent agreement, 35% good agreement, and 17% moderate agreement between the two assays for susceptible isolates and 100% excellent agreement for non-susceptible isolates. Overall, the assays compared provided good/fair statistical reproducibility for the assessment of phage susceptibility.

## Introduction

The escalating prevalence of antimicrobial-resistant bacterial infections has generated interest in alternative therapeutics. Lytic phages, biomodified phages, and/or purified lytic enzymes of phages are being considered to target bacteria at sites of infection. The effectiveness of personalized phage medicine hinges on the identification of the most suitable phage or phage combination to target a specific pathogen. The specificity of phages demands the assessment of several phages to address the diverse range of pathogenic bacteria (Pirnay et al., 2018; Suh et al., 2022). Despite the century-long history of phage therapy, the challenge of developing a validated diagnostic test for phage susceptibility testing (PST) that aligns with contemporary clinical requirements remains unresolved (Cui et al., 2019). PST is more complex than antibiotic susceptibility testing, as phages are intricate biological nanostructures composed of a variety of proteins and genetic material and subject to evolution. PST should ideally meet the requirements of high sensitivity and specificity, high throughput, random access (avoiding bulk or batch requirements), use of affordable materials and instruments, not requiring highly skilled technical staff, having short turnaround times, and delivering clear-cut standardized results. Unlike antimicrobial susceptibility testing, standardized, accurate, and reproducible PST methods, reported with validated interpretive criteria, are lacking (Evans et al., 2023). Moreover, the results used to assess the suitability of a particular phage for treating a specific pathogen should strive for simplicity and clarity. A comprehensive understanding of the precise origins of the variation in PST results and the underlying influences remains inadequately characterized.

Conventional PST may rely on a cascade of tests conducted on agar media to select a phage or phages against a particular bacterial target; a similar strategy is used to isolate new phages from environmental samples (i.e., enrichment of phage banks or phage hunting). Given the diverse sizes, shapes, and degrees of clearance exhibited by plaque assays, findings necessitate interpretation, thereby introducing potential for interpersonal variation and therefore subjectivity. Such intricate methods demand extensive hands-on time, overnight incubation, and the expertise of well-trained, highly skilled operators. Consequently, possibilities for automation and high throughput are somewhat restricted. Another drawback of plaque assays is the challenge of evaluating combinations of phages, bacteria, and antibiotics simultaneously. PST methods using liquid culture, on the other hand, offer the possibility of process automation and high throughput. Liquid assays comprise various forms of growth kinetic or metabolic detection assays that may utilize automated plate readers to provide potential real-time insight into phage activity by monitoring the impact on bacterial growth and metabolism. Liquid assays may take hours to days, depending on the bacterial species and experimental conditions selected. These assays may have limited detection thresholds, with standard optical density (OD) readers only being capable of detecting concentrations exceeding 1 × 10^7^ colony-forming units (CFUs)/ml [although solid surface assays may detect as few as 1 CFU per sample (Yerushalmy et al., 2023)]. Obstacles involved in rapidly testing a large number of phages are formidable, underscoring the need for standardized, high-throughput phage screening methods (Daubie et al., 2022). PST has been evaluated using wide-field lensless monitoring, surface plasmon resonance imaging, rapid hydrogel-based approaches, flow cytometry, confocal fluorescence microscopy (Low et al., 2020; Perlemoine et al., 2021; O'connell et al., 2022; Patpatia et al., 2022), the Biolog Omnilog^™^ instrument (Biolog Inc., Hayward, California) (Henry et al., 2012; Cunningham et al., 2022; Parmar et al., 2023), and OD measurements (Xie et al., 2018; Rajnovic et al., 2019).

**Figure 1:**
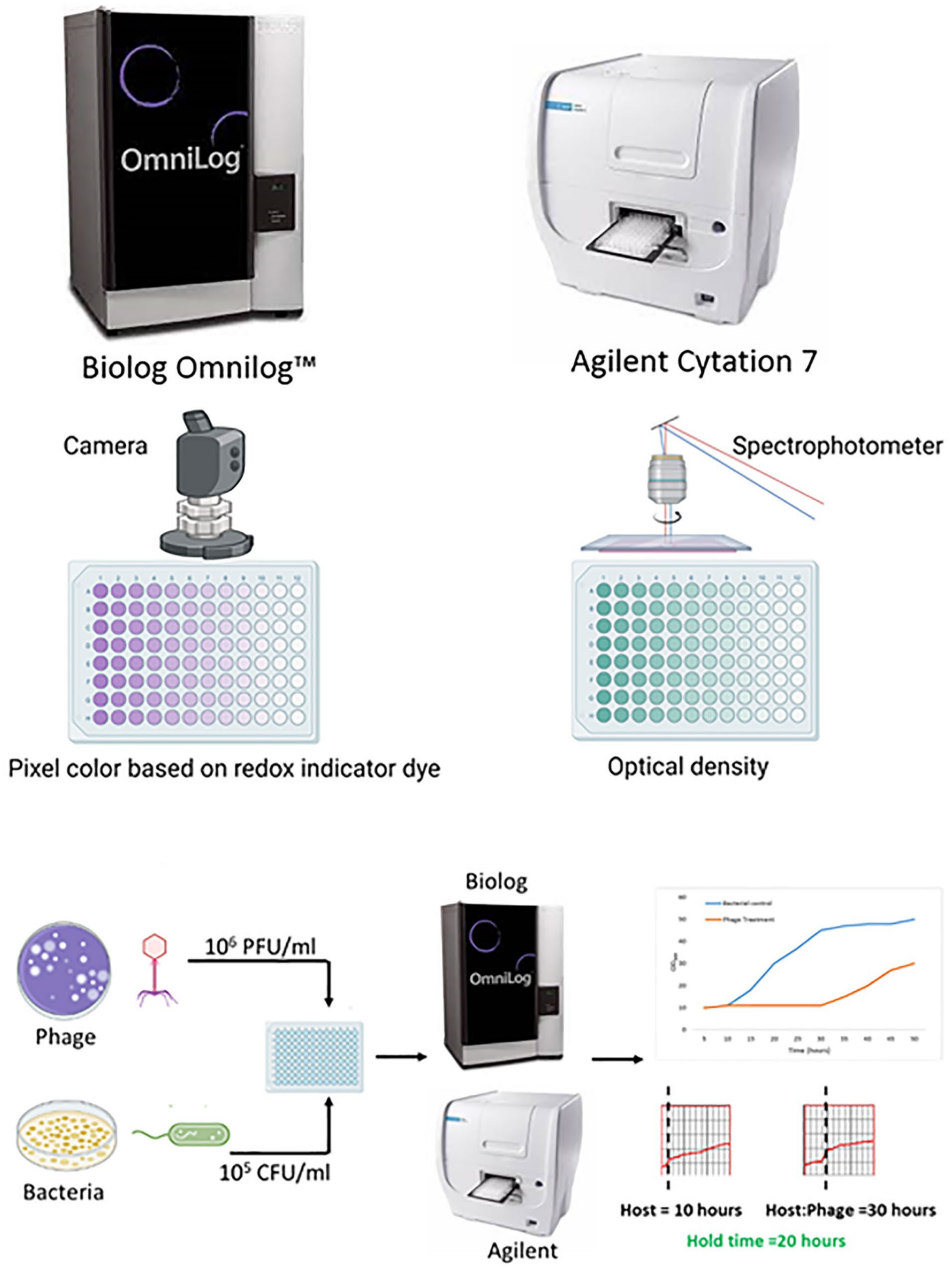
An overview of the comparison of phage susceptibility testing by two liquid high-throughput methods (created with Biorender.com).

Irrespective of the method used, the results must be reproducible within the method and agree with those of other methods. Herein, the reproducibility of PST performed on two high-throughput instruments, namely, the Biolog Omnilog^™^ and Agilent BioTeK Cytation 7 (Agilent Technologies Inc., Santa Clara, California), was evaluated (Figure 1). The Biolog Omnilog™ is a metabolic phenotyping system featuring an integrated digital camera system designed to quantify cellular respiration. The instrument accommodates up to 50 specialized Biolog 96-well microtiter plates, which can be stacked onto shelves in the instrument. The system utilizes a tetrazolium redox dye that undergoes color alteration correlating with cellular respiration. An integrated camera captures digital images at predetermined times throughout incubation. Colorimetric signals at individual time points are quantified in OmniLog units (OUs) and assessed on a scale spanning from 0 to 500. These values can be graphically represented, delivering a time-dependent curve. The instrument’s application in clinical and public health microbiology laboratories for phenotyping and antimicrobial susceptibility testing suggests potential suitability for clinical application (Cunningham et al., 2022; Vaillant et al., 2022). The Agilent BioTeK Cytation 7 represents a cell imaging multimode reader amalgamating automated digital upright and inverted widefield microscopy with multimode OD microplate reading capabilities. In the present study, a microplate reader furnished with an integrated spectrophotometer was used to read the OD at 600 nm.

## Materials and methods

Phage susceptibility was tested simultaneously on the Biolog Omnilog^™^ and Agilent BioTeK Cytation 7 instruments using 55 diverse phages against 13 *Escherichia coli*, 25 *Staphylococcus aureus*, 11 *Pseudomonas aeruginosa*, 3 *Acinetobacter baumannii,* and 3 *Enterococcus faecalis* isolates. Phages were tested against their respective susceptible bacterial hosts (with activity confirmed by plaque assay) in quintuplicate on five different days (except *P. aeruginosa* phages PaWRA01phi11, PaWRA01phi39, PaWRA02phi83, and PaWRA02phi87, which were tested in quintuplicate on the same day). A non-susceptible bacterial control for *E. coli*, *S. aureus*, *A. baumannii,* and *E. faecalis* phages, *P. aeruginosa* PaWRA01, was tested in triplicate on 3 different days. No non-susceptible bacterial control was studied for *P. aeruginosa* phages.

Preparation of 96-well assay plates for both assays was performed simultaneously using the same reagents, bacterial culture, and phage stock. The only difference was that for the Biolog Omnilog^™^ assay (Biolog assay), testing was performed in tetrazolium/TSB medium where trypticase soy broth (TSB, MilliporeSigma) was spiked with 1% (v/v) tetrazolium Dye D (Biolog, Inc.), while for the Agilent BioTeK Cytation 7 assay (Agilent assay), testing was performed in the TSB medium. Bacteria were grown in TSB on a shaker incubator at 37°C for 2 hours and standardized to OD_600_ 0.085–0.115 for bacterial inoculum preparation. Phages were stored at 4°C in glass bottles, which has been reported as a way to store phages to minimize loss in phage titer (Fortier and Moineau, 2009). Phages from stocks were quantified using a plaque assay and standardized to 10^8^ plaque-forming units (PFUs)/ml in phosphate-buffered saline. First, both plates were filled with appropriate medium—80 μL tetrazolium/TSB in the Biolog assay plate and 80 μL TSB in the Agilent assay plate—followed by 10 μL of bacteria (a final concentration of 10^5^ CFUs/ml) and 10 μL of phages (the final concentration of 10^6^ PFU/ml). Each plate included bacteria (10 μL of bacteria in 90 μL of medium), media (100 μL of medium), and phage (10 μL of phage in 90 μL of medium) controls. Each reagent in each well was added simultaneously to both assay plates.

Biolog assay plates were loaded onto the Biolog Omnilog^™^ instrument, and Agilent assay plates onto the Agilent BioTek Cytation 7 instrument. Assays were conducted at 37°C, with measurements taken every 15 min for 48 h. Omnilog Data Analysis Software (version 1.7, Biolog, Inc.) was used to analyze Biolog assay data; data from the Agilent assay were exported to Excel. PhageSelect^™^, a web-based software (Adaptive Phage Therapeutics Inc., USA), was used to calculate hold times (the time through which bacterial growth is inhibited by phage as compared to controls) for both assays. Hold times were evaluated based on the inflection time of phage and bacterial combinations compared to the inflection time of bacterial controls, with hold times ≥8 h considered indicative of active phage (Parmar et al., 2023), noting that there is no standardized breakpoint for defining phage activity. Studies performed in our laboratory using *S.aureus* showed substantial decreases in CFUs after 4 h of treatment with phages monitored over 48 h (data not shown).

Plaque spot assays were performed in triplicate on the same day to confirm the infectivity of phages; results were recorded as susceptible or non-susceptible based on observation or lack of observation of zones of clearance, respectively. Replicates resulting in less than 8 h hold times for susceptible hosts tested by liquid assays were excluded from the analysis.

Reproducibility of the Biolog and Agilent assays was compared for each assay type (intra-assay measurements) to determine the coefficient of variance (% CV), a relative gauge of variability, elucidating the magnitude of a standard deviation concerning its mean value. A % CV of ≤50% was considered indicative of a valid test. Reproducibility based on a hold time cutpoint of ≥8 h, indicative of a susceptible phage, was assessed, and the percentage of reproducible measurements between both systems was calculated for phages with their susceptible hosts, non-susceptible controls, and phage families. Agreement between the two assays was tested using the interclass correlation coefficient (ICC) (Bartko, 1966), an index comparing variability within a group to variability across groups used to test convergence or homogeneity of responses within groups. ICC values below 0.4 suggest a lack of substantial agreement, while those falling between 0.4 and 0.75 suggest moderate agreement. Values ranging from 0.75 to 0.9 suggest good agreement and values exceeding 0.90 represent a high degree (excellent) of agreement. ICC and %CV values were calculated with SAS version 9.4 (SAS Inc., Cary, NC).

## Results

**Figure 2:**
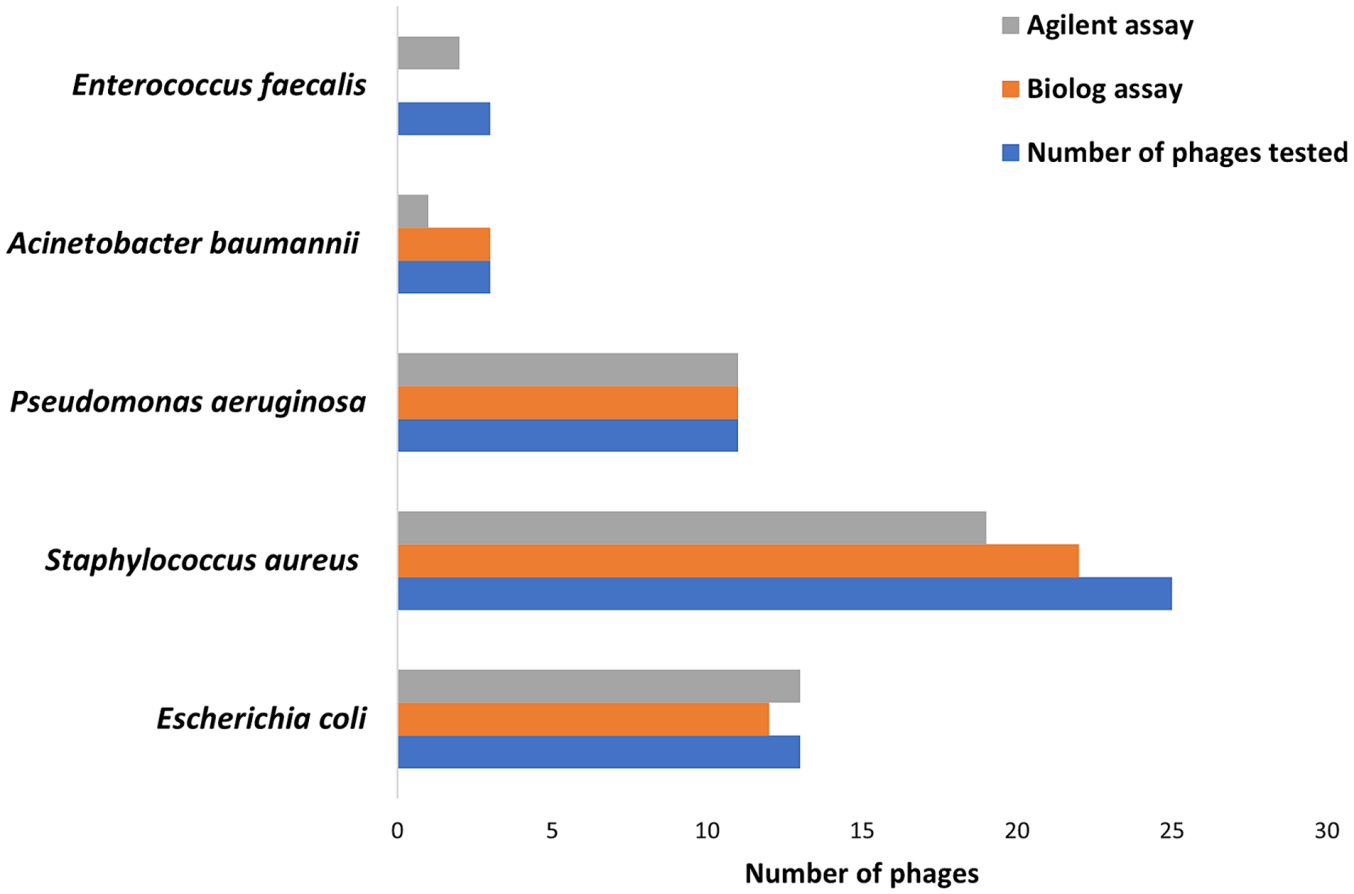
The number of phages showing valid results based on ≤50% coefficient of variance cutpoint for Biolog and Agilent assays against susceptible hosts.

Hold times for the two assays ranged from 0 to 48 h for 335 phage-bacteria combinations—including 203 susceptible phage/host combinations and 132 non-susceptible control assays. Scatterplots showing hold-time correlations between assays are shown in Supplementary Figures S1–S5. Means, standard deviations, and coefficients of variance (% CVs) were calculated for the hold times of each phage per assay (Table 1). Assays with non-susceptible controls showed no phage activity, and hence the hold times, means, standard deviations, and %CV were zero, while the ICC was one across all non-susceptible controls among both assays. A CV ≤50% was considered valid. A total of 92% Biolog and 90% Agilent assays yielded valid results for all intra-assay measurements. Among susceptible phage/host combinations, 87 and 84% were valid for the Biolog and Agilent assays, respectively, whereas for non-susceptible controls, 100% were valid for both assays. Based on different groups (Figure 2), *E. coli* phages showed 92% valid results by Biolog except for phage *EcCH06Phi7* and 100% valid results by Agilent. Staphylococcal phages resulted in 88% valid results by Biolog except for phages *SaRB105030Phi1*, *SCprASPhi1*, and *SCprJOPhi1*, which showed more than 50% CV; and 76% valid results by Agilent except for phages *SCprJOPhi1*, *S146406HNPhi1*, *S146406HNPhi2*, *S146406HPRIPhi1*, *S146406HPRIPhi2*, and *S146407HNPhi1*, which showed more than 50% CV, whereas *P. aeruginosa* phages resulted in 100% valid results by both Biolog and Agilent. *A. baumannii* phages resulted in 100% valid results by Biolog and 33% valid results by Agilent, except for phages *AbB2T9Phi4Ab2* and *AbB2T9PhiE1Ab3*, which showed more than 50% CV. The sample size for both *A. baumannii* and *E. faecalis* was small, with three phage/host combinations for each, leading to low %CVs and ICCs. *E. faecalis* phages yielded no valid results for Biolog, whereas, for Agilent, validity was 66% except for phage *VREPhi52Ef3*, which showed more than 50% CV. A %CV could not be measured for a few isolates because either the mean or standard deviation was zero.

**Table 1:** Hold time distribution of Biolog and Agilent assays.

Species	Phage	Host strain	Biolog			Agilent			ICC
			Mean hold time	SD	CV	Mean hold time	SD	CV	
**Susceptible phage/host combinations**									
*Escherichia coli*	EcCH06Phi7	EcCH06	27.5	23.3	84.9	11.5	2.1	18.4	0.3
	EcCH21Phi32	EcCH21	44.0	0.0	0.0	45.0	0.0	0.0	1.0
	EcCH24Phi48	EcCH24	36.3	15.5	42.8	35.5	17.7	49.8	−0.9
	EcCH26Phi37A	EcCH26	45.0	0.0	0.0	44.7	0.6	1.3	0.0
	EcCH27Phi38	EcCH27	8.5	0.7	8.3	19.0	7.1	37.2	−0.5
	EcCH29Phi40A	EcCH29	17.3	5.3	30.8	15.3	5.4	35.7	0.4
	EcCH31Phi42	EcCH31	27.3	13.3	48.7	31.5	13.0	41.3	−16.4
	EcCH32Phi43	EcCH32	44.5	0.7	1.6	45.5	0.7	1.6	1.0
	EcCH33Phi44	EcCH33	44.0	0.0	0.0	42.0	1.7	4.1	0.0
	EcCH36Phi47A	EcCH36	41.0	0.0	0.0	31.0	10.4	33.5	0.0
	EcCH56Phi56A	EcCH56	42.5	1.0	2.4	39.3	2.5	6.4	0.8
	EcCH61Phi61	EcCH61	46.0	1.4	3.1	43.5	0.7	1.6	0.9
	EcCH63Phi63A	EcCH63	43.5	0.7	1.6	44.0	0.0	0.0	0.0
*Staphylococcus aureus*	SaWIQ0456BPhi	SaWIQ0456B	44.0	1.7	3.9	43.0	1.4	3.3	0.9
	SaRB105030Phi1	SaRB105030	29.5	21.9	74.3	43.5	0.7	1.6	0.1
	SaRB105030Phi4	SaRB105030	43.8	1.9	4.3	44.3	1.0	2.2	0.9
	SaNS11469Phi1	SaNS11469	43.3	1.5	3.5	42.3	1.2	2.7	−20.0
	SaMD07Phi1	SaMD07	44.0	1.4	3.2	36.3	13.5	37.3	−0.2
	ScVDA3BHPhi1	ScVDA3BH	18.0	7.1	39.3	8.0	0.0	0.0	0.0
	ScVDA3ASMPhi1	ScVDA3ASM	12.2	1.6	13.5	14.4	3.9	27.2	0.1
	ScVDA3JFPhi1	ScVDA3JF	43.0	0.0	0.0	33.0	0.0	0.0	1.0
	SCprASPhi1	SCprAS	18.8	10.1	53.5	15.0	2.5	17.0	0.2
	SCprCWPhi4	SCprCW	42.0	0.0	0.0	38.0	0.0	0.0	1.0
	SCprGMPhi1	SCprGM	31.2	14.9	47.9	39.0	0.0	0.0	0.0
	SCprJOPhi1	SCprJO	19.3	15.5	80.6	21.0	11.5	54.6	1.0
	SCprMCFPhi2	SCprMCF	42.0	0.0	0.0	41.0	0.0	0.0	1.0
	SCprRPHPhi2	SCprRPH	43.0	0.0	0.0	14.0	5.7	40.4	0.0
	S146406HNPhi1	S146406HN	21.7	4.0	18.7	21.7	18.0	82.9	0.6
	S146406HNPhi2	S146406HN	20.7	1.2	5.6	22.0	17.8	80.8	0.2
	S146406HPRIPhi1	S146406HPRI	19.8	8.5	42.8	19.0	15.6	82.1	0.8
	S146406HPRIPhi2	S146406HPRI	12.5	3.5	28.3	12.5	6.4	50.9	−11.3
	S146406HPRIPhi3	S146406HPRI	45.0	0.0	0.0	17.0	0.0	0.0	1.0
	S146407FCPhi1	S146407FC	30.6	12.2	39.8	36.8	9.4	25.5	0.3
	S146407HNPhi1	S146407HN	25.6	12.2	47.7	43.0	40.5	94.2	−0.2
	SaWIQ0488Phi1	SaWIQ0488	38.4	9.2	24.0	24.4	11.1	45.5	−1.3
	S13SLPhi1	S13SL	37.0	9.6	25.9	38.8	11.6	30.0	0.8
	Se46386Phi4	Se46386	48.0	0.0	0.0	43.0	0.0	0.0	1.0
	SaMD22Phi1	SaMD22	12.2	1.5	12.2	42.0	0.0	0.0	0.0
*Acinetobacter baumannii*	AbB2T9Phi3AB1	AbB2T9	41.5	0.7	1.7	42.0	0.0	0.0	0.0
	AbB2T9Phi4Ab2	AbB2T9	33.0	14.7	44.6	21.3	18.1	85.1	0.7
	AbB2T9PhiE1Ab3	AbB2T9	32.3	15.0	46.4	32.7	17.0	52.2	1.0
*Enterococcus faecalis*	VRE25Phi1Ef1	VRE25	13.7	23.7	173.2	43.3	1.5	3.5	0.2
	VREPhi47Ef2	VRE27	28.0	24.3	86.7	43.3	1.5	3.5	0.0
	VREPhi52Ef3	VRE39	20.5	29.0	141.4	30.0	19.8	66.0	1.0
*Pseudomonas aeruginosa*	PaPhi15Pa1	PaT17875	31.8	15.2	47.8	37.8	3.2	8.5	0.6
	PaPhi16Pa2	PaT17875	31.8	10.4	32.7	31.8	11.6	36.5	−1.7
	PaPhi17Pa3	PaT17875	35.0	5.7	16.2	36.0	4.2	11.8	1.0
	PaPhi19Pa4	PaX30882	38.0	4.0	10.5	37.5	3.0	8.0	1.0
	PaPhi20Pa5	PaX30882	29.7	13.3	44.9	37.7	3.2	8.5	−0.1
	PaPhi26Pa6	PaT17875	26.2	12.6	48.1	21.8	9.8	45.1	0.6
	Pa14NPPhiPASA16Pa7	Pa14NP	41.5	1	2.4	40.8	1.5	3.7	1.0
	PaWRA01Phi11	PaWRA01	42.0	0.0	0.0	42.0	0.0	0.0	1.0
	PaWRA01Phi39	PaWRA01	26.3	6.4	24.4	23.0	1.6	7.1	−0.9
	PaWRA02Phi83	PaWRA02	18.6	4.2	22.7	13.8	2.6	18.8	−0.1
	PaWRA02Phi87	PaWRA02	18.2	3.7	20.3	14.8	1.5	10.0	0.1
**Non-susceptible phage/host combination controls**									
*E. coli*	EcCH06Phi7	PaWRA01	0	0	0	0	0	0	1
	EcCH21Phi32	PaWRA01	0	0	0	0	0	0	1
	EcCH24Phi48	PaWRA01	0	0	0	0	0	0	1
	EcCH26Phi37A	PaWRA01	0	0	0	0	0	0	1
	EcCH27Phi38	PaWRA01	0	0	0	0	0	0	1
	EcCH29Phi40A	PaWRA01	0	0	0	0	0	0	1
	EcCH31Phi42	PaWRA01	0	0	0	0	0	0	1
	EcCH32Phi43	PaWRA01	0	0	0	0	0	0	1
	EcCH33Phi44	PaWRA01	0	0	0	0	0	0	1
	EcCH36Phi47A	PaWRA01	0	0	0	0	0	0	1
	EcCH56Phi56A	PaWRA01	0	0	0	0	0	0	1
	EcCH61Phi61	PaWRA01	0	0	0	0	0	0	1
	EcCH63Phi63A	PaWRA01	0	0	0	0	0	0	1
*S. aureus*	SaWIQ0456BPhi	PaWRA01	0	0	0	0	0	0	1
	SaRB105030Phi1	PaWRA01	0	0	0	0	0	0	1
	SaRB105030Phi4	PaWRA01	0	0	0	0	0	0	1
	SaNS11469Phi1	PaWRA01	0	0	0	0	0	0	1
	SaMD07Phi1	PaWRA01	0	0	0	0	0	0	1
	ScVDA3BHPhi1	PaWRA01	0	0	0	0	0	0	1
	ScVDA3ASMPhi1	PaWRA01	0	0	0	0	0	0	1
	ScVDA3JFPhi1	PaWRA01	0	0	0	0	0	0	1
	SCprASPhi1	PaWRA01	0	0	0	0	0	0	1
	SCprCWPhi4	PaWRA01	0	0	0	0	0	0	1
	SCprGMPhi1	PaWRA01	0	0	0	0	0	0	1
	SCprJOPhi1	PaWRA01	0	0	0	0	0	0	1
	SCprMCFPhi2	PaWRA01	0	0	0	0	0	0	1
	SCprRPHPhi2	PaWRA01	0	0	0	0	0	0	1
	S146406HNPhi1	PaWRA01	0	0	0	0	0	0	1
	S146406HNPhi2	PaWRA01	0	0	0	0	0	0	1
	S146406HPRIPhi1	PaWRA01	0	0	0	0	0	0	1
	S146406HPRIPhi2	PaWRA01	0	0	0	0	0	0	1
	S146406HPRIPhi3	PaWRA01	0	0	0	0	0	0	1
	S146407FCPhi1	PaWRA01	0	0	0	0	0	0	1
	S146407HNPhi1	PaWRA01	0	0	0	0	0	0	1
	SaWIQ0488Phi1	PaWRA01	0	0	0	0	0	0	1
	S13SLPhi1	PaWRA01	0	0	0	0	0	0	1
	Se46386Phi4	PaWRA01	0	0	0	0	0	0	1
	SaMD22Phi1	PaWRA01	0	0	0	0	0	0	1
*A. baumannii*	AbB2T9Phi3Ab1	PaWRA01	0	0	0	0	0	0	1
	AbB2T9Phi4Ab2	PaWRA01	0	0	0	0	0	0	1
	AbB2T9PhiE1AB3	PaWRA01	0	0	0	0	0	0	1
*E. faecalis*	VRE25Phi1Ef1	PaWRA01	0	0	0	0	0	0	1
	VREPhi47Ef2	PaWRA01	0	0	0	0	0	0	1
	VREPhi52Ef3	PaWRA01	0	0	0	0	0	0	1

SD, Standard deviation; CV, coefficient of variance percent; ICC, interclass correlation coefficient.

ICC values showed that 26% of phages (15 phages) yielded results in excellent agreement; these phages were *EcCH21Phi32*, *EcCH32Phi43*, *SaWIQ0456BPhi*, *ScVDA3JFPhi1*, *SCprCWPhi4*, *SCprJOPhi1*, *SCprMCFPhi2*, *S146406HPRIPhi3*, *Se46386Phi4*, *AbB2T9PhiE1Ab3*, *VREPhi52Ef3*, *PaPhi17Pa3*, *PaPhi19Pa4*, *Pa14NPPhiPASA16Pa7,* and *PaWRA01Phi11*. A total of 35% of phages (20 phages) yielded results in good agreement and 17% (10 phages) in moderate agreement for inter-assay reproducibility. Phages *EcCH24Phi48*, *EcCH27Phi38*, *EcCH31Phi42*, *SaNS11469Phi1*, *SaMD07Phi1*, *S146406HPRIPhi2*, *S146407HNPhi1*, *SaWIQ0488Phi1*, *PaPhi16Pa2*, *PaPhi20Pa5,* and *PaWRA01Phi39* resulted in negative ICC values, indicating that intra-assay variability exceeded inter-assay variability. The small sample size likely contributed to the decrease in ICC. There was a complete correlation between the results of the two study assays and the plaque assays (Table 1).

## Discussion

Phage therapy holds potential as a treatment for persistent infections, but some parameters informing its potential user need further definition. Comprehensive scientific inquiries are essential to bridge significant knowledge gaps prior to routine adoption of phage therapy in clinical practice. Phage clinical trials are needed to address the safety and efficacy of phages as antimicrobial agents (Tamma et al., 2022). Phages are intricate genetic entities that undergo replication and frequent mutation. Their activity is influenced by a multitude of factors, including the specific bacterial host and prevailing environmental conditions (Storms et al., 2020). While there has been growing acceptance that phages should be precisely matched to their target bacteria, a lack of PST standardization complicates the ability to compare and study phage therapy treatments.

Here, we compared the reproducibility of PST between two liquid high-throughput methods, namely, the Biolog Omnilog^™^ and the Agilent BioTeK Cytation 7 instruments. Measurements were performed in quintuplicate for susceptible phage–host pairs and in triplicate for non-susceptible controls, with both assays performed simultaneously. Using an 8-h hold time cutpoint, all runs by both assays were in categorical agreement. There was, however, variability in individual hold times within and between the platforms. Based on a ≤50% CV cutpoint, 92 and 90% of the results were valid for the Biolog and Agilent assays, respectively. More than half of the variance could have been related to differences between replicate tests conducted on different days and the two assay types. The remainder of the variability was likely biological, involving either or both bacteria and phages.

While the reported studies were performed using the same reagents, and plates were simultaneously prepared for both assays, replicate testing was performed on different days, possibly introducing variability in phage stock and/or bacterial inoculum. Bacteria were standardized to an OD_600_ of 0.085–0.115. The OD does not necessarily, however, account for dead cells or aggregates (Cooper et al., 2011; Rajnovic et al., 2019). Inoculum preparation of the same bacterial species by the same person on different days has been reported to be associated with 7–25% variation (Hombach et al., 2016). The results may be compounded by diverse environmental factors, including subtle disparities in culture media, phage storage conditions, procedural setup, or incubation temperatures. Repeat measurements are also prone to divergent outcomes owing to inherent variability in cellular populations across cultures, stemming from natural diversity. Notably, biological variance also contributes to variability in antibiotic susceptibility testing (Hombach et al., 2016).

The same number of phages was used for replicate experiments performed on different days. Phages were stored at 4°C in glass containers and may have attached to glass surfaces, lowering actual titers of phage assayed. Phage tail structures may break while pipetting and mixing, which may lead to a decrease in overall phage numbers. Another complexity is that bacteria may develop resistance to phages through mutation and employ a diverse array of mechanisms to shield themselves against phage infections, including (1) loss or alteration of phage receptors, (2) prevention of phage adsorption, (3) prevention of infection by restriction-modification, (4) degradation of phage genetic material/blocking replication, and (5) cell death, thereby protecting nearby cells from further infections (Koskella and Brockhurst, 2014). This may lower the reproducibility of PST. The selection of media may impact on PST, as phages may behave differently based on their microenvironment. The same lot of TSB medium was used for all assays, with tetrazolium/TSB prepared freshly every day. This may have introduced variability, resulting in differences in hold times owing to changes in areas under the curve as detected by the Biolog camera.

The present study initially included replicates of each phage; however, samples that failed quality checks were excluded, reducing the sample size. The small sample size may have led to a decrease in ICC, affecting the overall results. ICC values showed 26% of results to be in excellent agreement, 35% to be in good agreement, and 17% to be in moderate agreement. The ICC can be low or negative, indicating poor agreement when variability within groups exceeds variability across groups. This means that differences in measurements performed with the same assay type (i.e., either Biolog or Agilent) on different days were higher compared to differences between measurements of the two assays performed on the same day. It is normal for the ICC to be −1 for dyads (sample size of two) (Taylor, 2010; Costa-Santos et al., 2011).

While experimental reagents were standardized to maximize reproducibility, differences in the working principles of both assays can result in differences in hold times among replicates. The Biolog Omnilog^™^ system detects alterations in dye color captured by a camera, while the Agilent BioTeK Cytation 7 system detects variations in absorbance measured by a spectrophotometer. In the Biolog assay, the development of color, based on metabolism, can take time after the actual decrease in cell death. This may result in hold time differences compared to the Agilent assay. The Omnilog has lower sensitivity to bacterial aggregates compared to absorbance-based systems. The Omnilog cannot differentiate dead or non-metabolizing cells from live cells and has limited detection capacity for late lysis after growth (Henry et al., 2012; Cunningham et al., 2022; Yerushalmy et al., 2023), although it has been suggested that the signal intensity detected and measured by the Omnilog’s camera is satisfactory for antibacterial activity assays (Cruz et al., 2021). The same report suggested non-significant variability in antibiotic minimum inhibitory concentration values when compared to a conventional method of antibiotic susceptibility testing. Agilent absorbance measurements offer simplicity in execution but exhibit lower sensitivity and validity within a constrained concentration range.

Apart from issues relating to methodology, no standardized breakpoints for PST have been defined to differentiate sensitive/active from non-sensitive/inactive phage activity. In this study, a hold time cutpoint of 8 h was applied (Parmar et al., 2023). Categorical attributes based on plaque assays may be used to elucidate the activity of phages; quantitative values assessing the correlation between specific hold times or areas under the curve, and attributes leading to *in vivo* phage activity are poorly defined. While validated standards are lacking, phage therapy centers worldwide have suggested various PST breakpoints based on the clinical and compassionate use of phage therapy (Pirnay et al., 2018; Daubie et al., 2022; Yerushalmy et al., 2023).

In conclusion, the reproducibility of PST among the two assays revealed good agreement, but the low ICCs suggest differences among measurements on different days to be fair as compared to same-day measurements performed by the two assay types—the Biolog Omnilog^™^ and Agilent BioTeK Cytation 7. Estimations of variability reasonably mirror daily practice and encompass technical variations. Considering that interactions among the two biological entities—phages and bacteria—are influenced by various environmental factors, defining stable testing conditions will be necessary to further standardize PST. Progress in PST will drive phage therapy toward a potential precision treatment and allow future assessment of engineered phages and phage cocktails using expanded methods.

## Data availability statement

The original contributions presented in the study are included in the article/Supplementary material, further inquiries can be directed to the corresponding author.

## Author contributions

KP: Conceptualization, Formal analysis, Methodology, Writing – original draft, Writing – review & editing. JF: Project administration, Resources, Validation, Writing – review & editing. ZR: Project administration, Resources, Validation, Writing – review & editing. JM: Data curation, Formal analysis, Writing – review & editing. KG-Q: Project administration, Supervision, Writing – review & editing. RP: Conceptualization, Funding acquisition, Supervision, Writing – original draft, Writing – review & editing.

## References

[ref1] Bartko J. J. (1966). The intraclass correlation coefficient as a measure of reliability. Psychol. Rep. 19, 3–11. doi: 10.2466/pr0.1966.19.1.3 5942109

[ref2] Cooper C. J. Denyer S. P. Maillard J. Y. (2011). Rapid and quantitative automated measurement of bacteriophage activity against cystic fibrosis isolates of *Pseudomonas aeruginosa*. J. Appl. Microbiol. 110, 631–640. doi: 10.1111/j.1365-2672.2010.04928.x, PMID: 21205097

[ref3] Costa-Santos C. Bernardes J. Ayres-De-Campos D. Costa A. Costa C. (2011). The limits of agreement and the intraclass correlation coefficient may be inconsistent in the interpretation of agreement. J. Clin. Epidemiol. 64, 264–269. doi: 10.1016/j.jclinepi.2009.11.010, PMID: 20189765

[ref4] Cruz C. D. Esteve P. Tammela P. (2021). Evaluation and validation of Biolog OmniLog® system for antibacterial activity assays. Appl. Microbiol. 72, 589–595. doi: 10.1111/lam.13450, PMID: 33428794

[ref5] Cui Z. L. Guo X. K. Feng T. T. Li L. (2019). Exploring the whole standard operating procedure for phage therapy in clinical practice. J. Transl. Med. 17, 1–7. doi: 10.1186/s12967-019-2120-z 31727099 PMC6857313

[ref6] Cunningham S. A. Mandrekar J. N. Suh G. Patel R. (2022). Preliminary reproducibility evaluation of a phage susceptibility testing method using a collection of *Escherichia coli* and *Staphylococcus aureus* phages. J. Appl. Lab Med. 7, 1468–1475. doi: 10.1093/jalm/jfac051 35818639

[ref7] Daubie V. Chalhoub H. Blasdel B. Dahma H. Merabishvili M. Glonti T.. (2022). Determination of phage susceptibility as a clinical diagnostic tool: A routine perspective. Front. Cell. Infect. Microbiol. 12:1000721. doi: 10.3389/fcimb.2022.1000721, PMID: 36211951 PMC9532704

[ref8] Evans S. R. Patel R. Hamasaki T. Howard-Anderson J. Kinamon T. King H. A.. (2023). The future ain’t what it used to be… Out with the old… In with the better: Antibacterial Resistance Leadership Group innovations. Clin. Infect. Dis. 77, S321–S330. doi: 10.1093/cid/ciad538, PMID: 37843122 PMC10578048

[ref9] Fortier L. C. Moineau S. (2009). Phage production and maintenance of stocks, including expected stock lifetimes. Methods Mol. Biol. 501, 203–219. doi: 10.1007/978-1-60327-164-6_19 19066823

[ref10] Henry M. Biswas B. Vincent L. Mokashi V. Schuch R. Bishop-Lilly K. A.. (2012). Development of a high throughput assay for indirectly measuring phage growth using the OmniLog™ system. Bacteriophage 2, 159–167. doi: 10.4161/bact.21440, PMID: 23275867 PMC3530525

[ref11] Hombach M. Ochoa C. Maurer F. P. Pfiffner T. Bottger E. C. Furrer R. (2016). Relative contribution of biological variation and technical variables to zone diameter variations of disc diffusion susceptibility testing. J. Antimicrob. Chemother. 71, 141–151. doi: 10.1093/jac/dkv309, PMID: 26462987

[ref12] Koskella B. Brockhurst M. A. (2014). Bacteria-phage coevolution as a driver of ecological and evolutionary processes in microbial communities. FEMS Microbiol. Rev. 38, 916–931. doi: 10.1111/1574-6976.12072, PMID: 24617569 PMC4257071

[ref13] Low H. Z. Böhnlein C. Sprotte S. Wagner N. Fiedler G. Kabisch J.. (2020). Fast and easy phage-tagging and live/dead analysis for the rapid monitoring of bacteriophage infection. Front. Microbiol. 11:602444. doi: 10.3389/fmicb.2020.602444, PMID: 33391221 PMC7775415

[ref14] O'connell L. Mandula O. Leroy L. Aubert A. Marcoux P. R. Roupioz Y. (2022). Ultrafast and multiplexed bacteriophage susceptibility testing by surface plasmon resonance and phase imaging of immobilized phage microarrays. Chemosensors 10:192. doi: 10.3390/chemosensors10050192

[ref15] Parmar K. Komarow L. Ellison D. W. Filippov A. A. Nikolich M. P. Fackler J. R.. (2023). Interlaboratory comparison of *Pseudomonas aeruginosa* phage susceptibility testing. J. Clin. Microbiol. 61, e00614–e00623. doi: 10.1128/jcm.00614-23 37962552 PMC10729752

[ref16] Patpatia S. Schaedig E. Dirks A. Paasonen L. Skurnik M. Kiljunen S. (2022). Rapid hydrogel-based phage susceptibility test for pathogenic bacteria. Front. Cell. Infect. Microbiol. 12:1829. doi: 10.3389/fcimb.2022.1032052 PMC9771388 36569196

[ref17] Perlemoine P. Marcoux P. R. Picard E. Hadji E. Zelsmann M. Mugnier G.. (2021). Phage susceptibility testing and infectious titer determination through wide-field lensless monitoring of phage plaque growth. PLoS One 16:e0248917. doi: 10.1371/journal.pone.0248917, PMID: 33755710 PMC7987195

[ref18] Pirnay J. P. Verbeken G. Ceyssens P. J. Huys I. De Vos D. Ameloot C.. (2018). The Magistral Phage. Viruses 10:64. doi: 10.3390/v10020064 29415431 PMC5850371

[ref19] Rajnovic D. Muñoz-Berbel X. Mas J. (2019). Fast phage detection and quantification: An optical density-based approach. PLoS One 14:e0216292. doi: 10.1371/journal.pone.0216292, PMID: 31071103 PMC6508699

[ref20] Storms Z. J. Teel M. R. Mercurio K. Sauvageau D. (2020). The virulence index: A metric for quantitative analysis of phage virulence. Phage 1, 27–36. doi: 10.1089/phage.2019.0001, PMID: 36147620 PMC9041455

[ref21] Suh G. A. Lodise T. P. Tamma P. D. Knisely J. M. Alexander J. Aslam S.. (2022). Considerations for the use of phage therapy in clinical practice. Antimicrob. Agents Chemother. 66:e02071. doi: 10.1128/aac.02071-21 35041506 PMC8923208

[ref22] Tamma P. D. Souli M. Billard M. Campbell J. Conrad D. Ellison D. W.. (2022). Safety and microbiological activity of phage therapy in persons with cystic fibrosis colonized with *Pseudomonas aeruginosa*: Study protocol for a phase 1b/2, multicenter, randomized, double-blind, placebo-controlled trial. Trials 23:1057. doi: 10.1186/s13063-022-07047-5, PMID: 36578069 PMC9795609

[ref23] Taylor P. J. (2010). An introduction to intraclass correlation that resolves some common confusions. Unpublished manuscript, vol. 4. Boston: University of Massachusetts, 137–155.

[ref24] Vaillant J. J. Cunningham S. A. Patel R. (2022). Antibiotic susceptibility testing of *Staphylococcus aureus* using the Biolog OmniLog® system, a metabolic phenotyping assay. Diagn. Microbiol. Infect. Dis. 104:115759. doi: 10.1016/j.diagmicrobio.2022.115759, PMID: 35872370

[ref25] Xie Y. Wahab L. Gill J. J. (2018). Development and validation of a microtiter plate-based assay for determination of bacteriophage host range and virulence. Viruses 10:189. doi: 10.3390/v10040189, PMID: 29649135 PMC5923483

[ref26] Yerushalmy O. Braunstein R. Alkalay-Oren S. Rimon A. Coppenhagn-Glazer S. Onallah H.. (2023). Towards standardization of phage susceptibility testing: The Israeli phage therapy center “Clinical Phage Microbiology”—A pipeline proposal. Clin. Infect. Dis. 77, S337–S351. doi: 10.1093/cid/ciad514 37932122

