# Supplementary material for: A comparison of phage susceptibility testing with two liquid high-throughput methods
Source: Front Microbiol. 2024 Aug 7;15:1386245. doi: 10.3389/fmicb.2024.1386245 (PMC11335653; doi:10.3389/fmicb.2024.1386245)
Supplement: Supplementary file 1 [file Data_Sheet_1.pdf]

Comparison of Phage Susceptibility Testing by Two Liquid High-Throughput Methods

Krupa Parmar[1], Joseph R. Fackler[2], Zuriel Rivas[2], Jay Mandrekar[3],

Kerryl E. Greenwood-Quaintance[1], Robin Patel[1,4*]

[1]Division of Clinical Microbiology, Department of Laboratory Medicine and Pathology, Mayo Clinic, Rochester, MN, USA

[2]Adaptive Phage Therapeutics, Inc. (APT), Gaithersburg, MD, USA

[3]Division of Biomedical Statistics and Informatics, Department of Health Sciences Research, Mayo Clinic, Rochester, MN, USA

[4]Division of Public Health, Infectious Diseases, and Occupational Medicine, Department of Medicine, Mayo Clinic, Rochester, MN, USA

***Corresponding author**

Robin Patel, M.D.

Division of Clinical Microbiology, Mayo Clinic,

200 First Street SW, Rochester, MN 55905

Phone - 507-538-0579

Fax - 507-284-4272

email: patel.robin@mayo.edu

Supplementary Figures

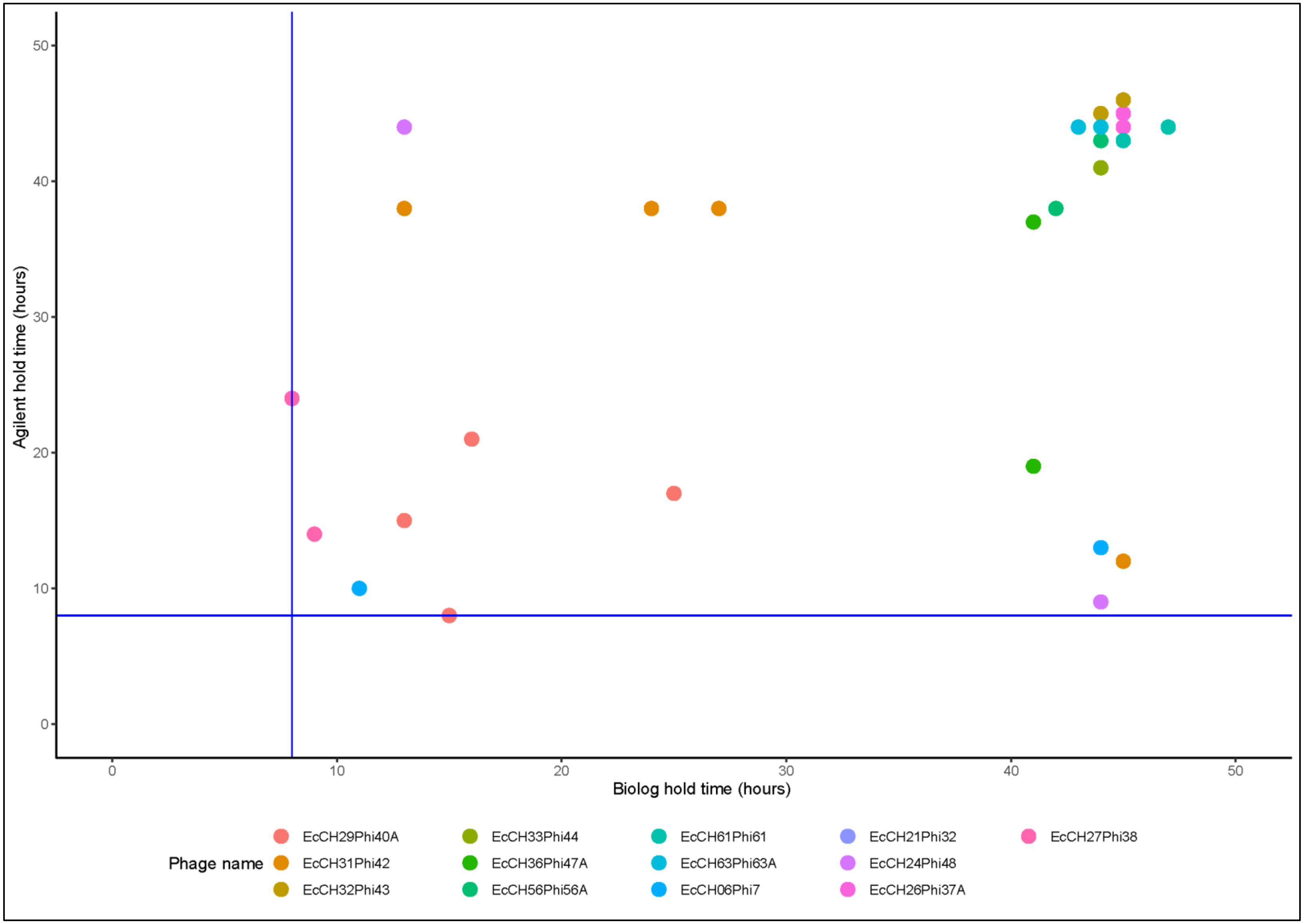

Supplementary Fig. 1 Scatterplots for hold time distribution of Biolog Omnilog™ and Agilent BioTek Cytation 7 assays for *Escherichia coli* phages (replicates) - EcCH06Phi7 (2), EcCH21Phi32 (4), EcCH24Phi48 (4), EcCH26Phi37A (3), EcCH27Phi38 (2), EcCH29Phi40A (4), EcCH31Phi42 (4), EcCH32Phi43 (2), EcCH33Phi44 (3), EcCH36Phi47A (3), EcCH56Phi56A (4), EcCH61Phi61 (2) and EcCH63Phi63A (2) against *E. coli* hosts. (Blue line indicates 8-hour hold time cutpoint.)

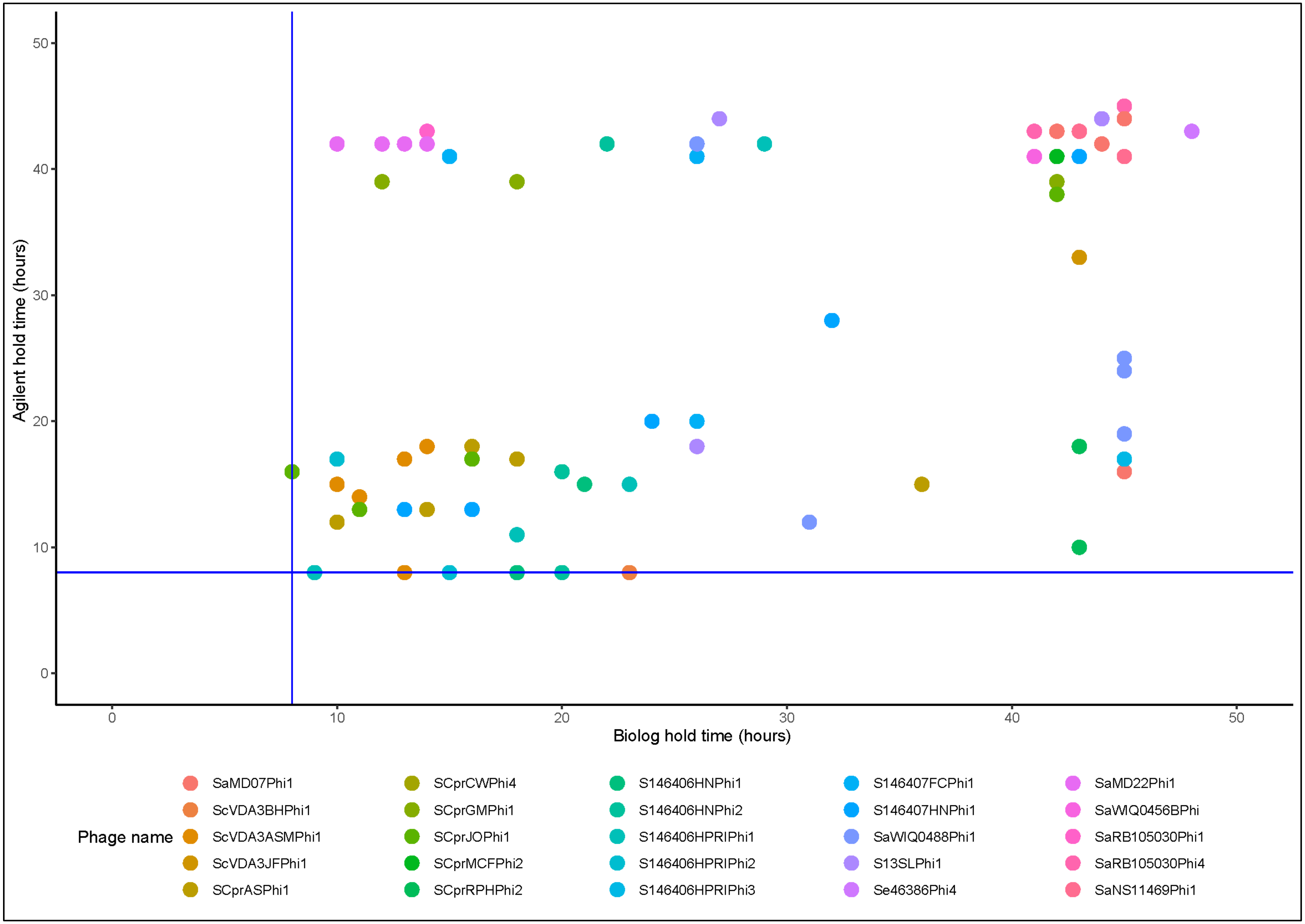

Agilent hold time (hours)
50
40
30
20
10
0
0
10
20
30
40
50
Biolog hold time (hours)
Phage name
SaMD07Phi1
ScVDA3BHPhi1
ScVDA3ASMPhi1
ScVDA3JFPhi1
SCprASPhi1
SCprCWPhi4
SCprGMPhi1
SCprJOPhi1
SCprMCFPhi2
SCprRPHPhi2
S146406HNPhi1
S146406HNPhi2
S146406HPRIPhi1
S146406HPRIPhi2
S146406HPRIPhi3
S146407FCPhi1
S146407HNPhi1
SaWIQ0488Phi1
S13SLPhi1
Se46386Phi4
SaMD22Phi1
SaWIQ0456BPhi
SaRB105030Phi1
SaRB105030Phi4
SaNS11469Phi1

Supplementary Fig. 2 Scatterplots for hold time distribution of Biolog Omnilog™ and Agilent BioTek Cytation 7 assays for *Staphylococcus aureus* phages (replicates) - SaWIQ0456BPhi (5), SaRB105030Phi1 (2), SaRB105030Phi4 (4), SaNS11469Phi1 (3), SaMD07Phi1 (4), ScVDA3BHPhi1 (2), ScVDA3ASMPhi1 (5), ScVDA3JFPhi1 (5), SCprASPhi1 (5), SCprCWPhi4 (5), SCprGMPhi1 (5), SCprJOPhi1 (4), SCprMCFPhi2 (5), SCprRPHPhi2 (2), S146406HNPhi1 (3), S146406HNPhi2 (3), S146406HPRIPhi1 (4), S146406HPRIPhi2 (2), S146406HPRIPhi3 (4), S146407FCPhi1 (5), S146407HNPhi1 (5), SaWIQ0488Phi1 (5), S13SLPhi1 (5), Se46386Phi4 (5) and SaMD22Phi1 (5) against *S. aureus* hosts. (Blue line indicates 8-hour hold time cutpoint.)

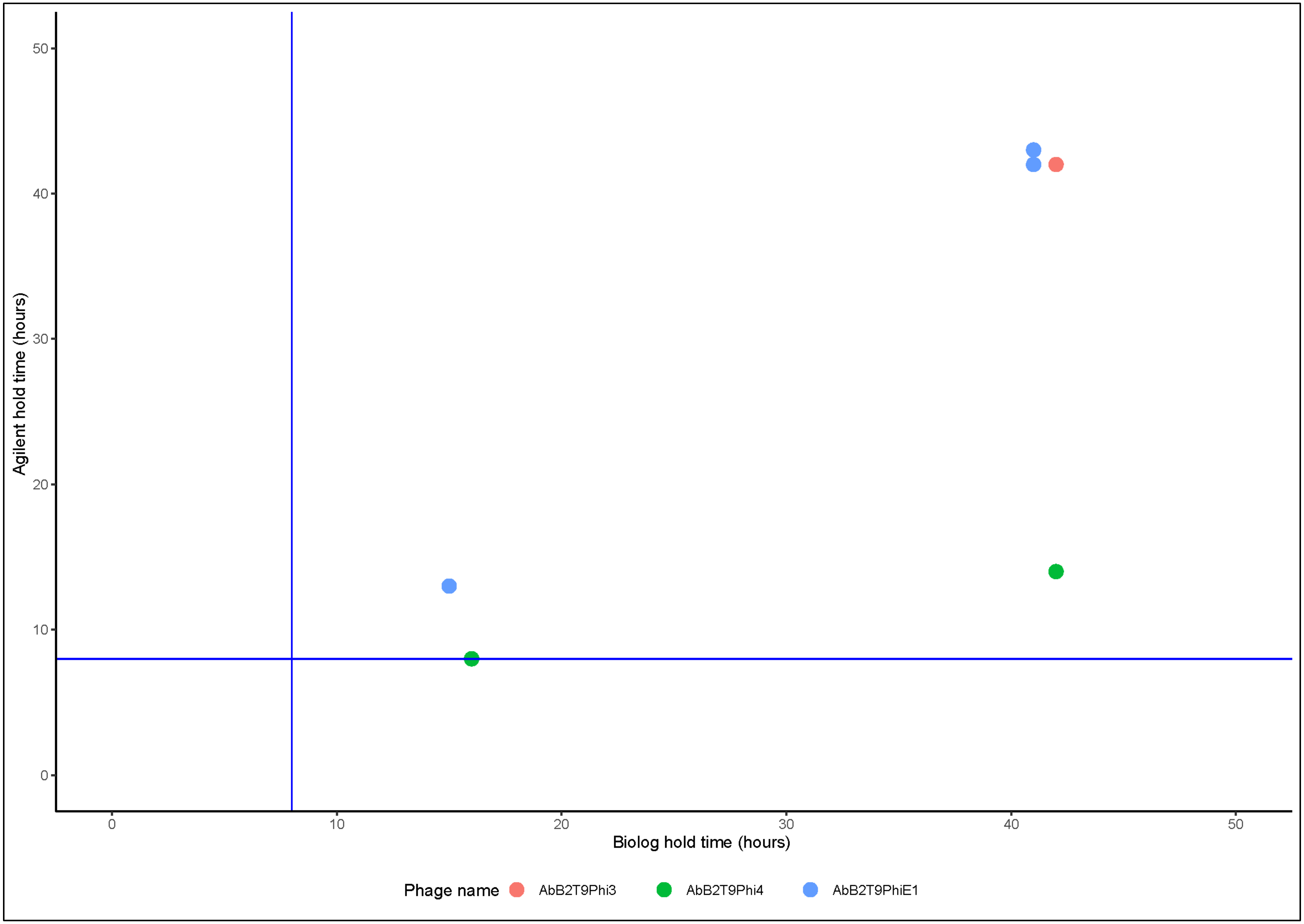

Agilent hold time (hours)
0
10
20
30
40
50
Biolog hold time (hours)
Phage name
AbB2T9Phi3
AbB2T9Phi4
AbB2T9PhiE1

Supplementary Fig. 3 Scatterplots for hold time distribution of Biolog Omnilog™ and Agilent BioTek Cytation 7 assays for *Acinetobacter baumannii* phages (replicates) - AbB2T9Phi3 (2), AbB2T9Phi4 (3) and AbB2T9PhiE1 (3) against *A. baumannii* host AbB2T9. (Blue line indicates 8-hour hold time cutpoint.)

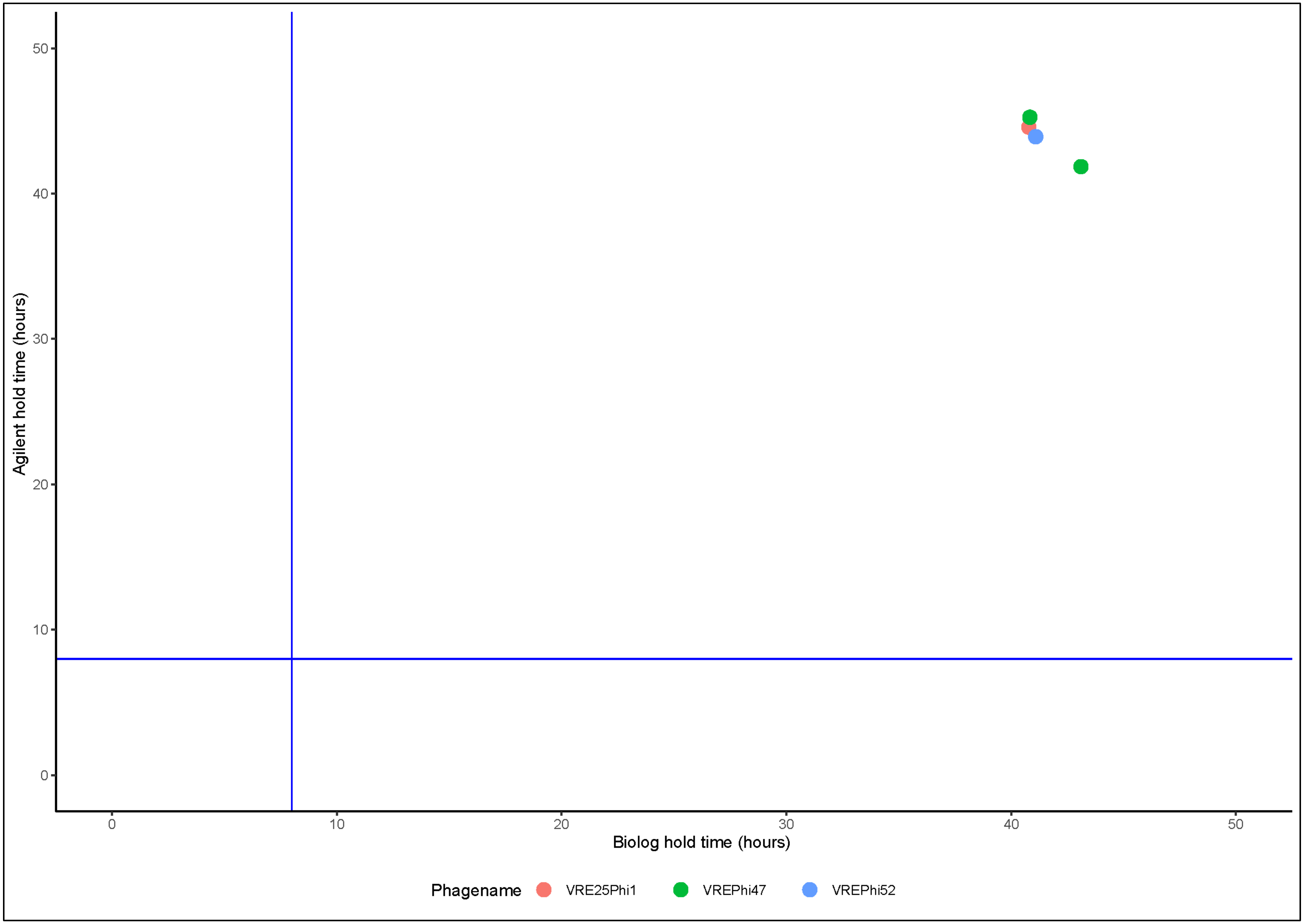

Agilent hold time (hours)
0
10
20
30
40
50
Biolog hold time (hours)
Phagename
VRE25Phi1
VREPhi47
VREPhi52

Supplementary Fig. 4 Scatterplots for hold time distribution of Biolog Omnilog™ and Agilent BioTek Cytation 7 assays for *Enterococcus faecalis* phages (replicates) - VRE25Phi1 (3), VREPhi47 (3) and VREPhi52 (2) against *E. faecalis* hosts. (Blue line indicates 8-hour hold time cutpoint.)

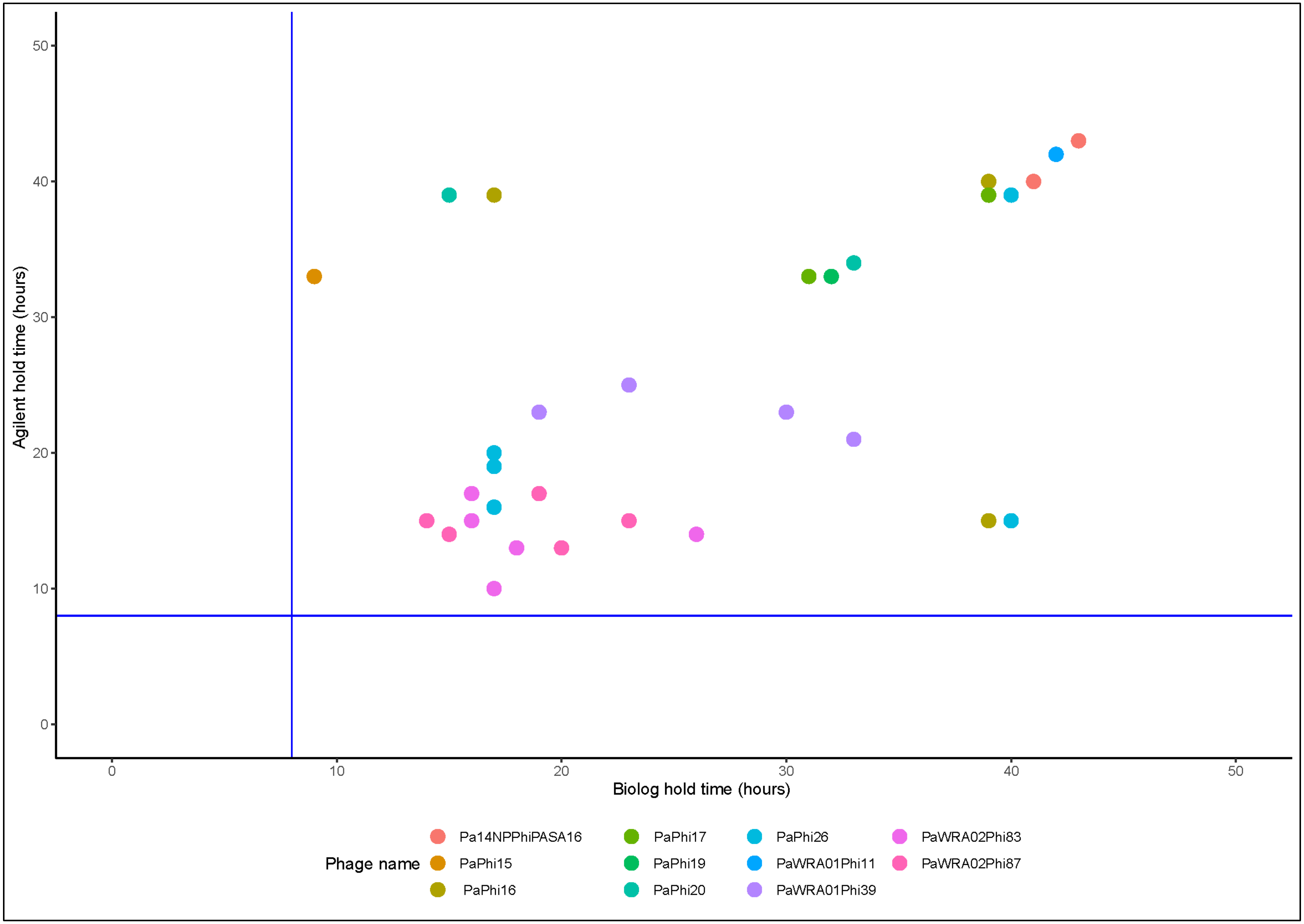

Agilent hold time (hours)
0
10
20
30
40
50
Biolog hold time (hours)
0
10
20
30
40
50
Phage name
Pa14NPPhiPASA16
PaPhi15
PaPhi16
PaPhi17
PaPhi19
PaPhi20
PaPhi26
PaWRA01Phi11
PaWRA01Phi39
PaWRA02Phi83
PaWRA02Phi87

Supplementary Fig. 5 Scatterplots for hold time distribution of Biolog Omnilog™ and Agilent BioTek Cytation 7 assays for *Pseudomonas aeruginosa* phages (replicates) - PaPhi15 (4), PaPhi16 (4), PaPhi17 (2), PaPhi19 (4), PaPhi20 (3), PaPhi26 (5), Pa14NPPhiPASA16 (4), PaWRA01Phi11 (4), PaWRA01Phi39 (4), PaWRA02Phi83 (5) and PaWRA02Phi87 (5) against *P. aeruginosa* hosts. (Blue line indicates 8-hour hold time cutpoint.)
